# Community engagement to tackle infectious threats: A viewpoint based on a social science mapping process in Bangladesh, Uganda, and Ukraine

**DOI:** 10.7189/jogh.13.03025

**Published:** 2023-06-23

**Authors:** Elena Jirovsky-Platter, Paul Grohma, Nahitun Naher, Roman Rodyna, Christine Nabirye, Michel Dückers, Syed M Ahmed, Jacob Osborne, David Kaawa-Mafigiri, Tamara Giles-Vernick, Ruth Kutalek

**Affiliations:** 1 Medical University of Vienna, Vienna, Austria; 2 BRAC University, Dhaka, Bangladesh; 3 PATH, project officer, COVID-19 response, Ukraine; 4 Makerere University, Kampala, Uganda; 5 Nederlands instituut voor onderzoek van de gezondheidszorg – NIVEL, Utrecht, the Netherlands; 6 Institut Pasteur, Université Paris Cité, Paris, France

The SoNAR-Global Social Science Network for Infectious Threats and Antimicrobial Resistance [1] connects social scientists from Europe, South East Asia, and West and Central Africa in their interest in epidemics and infectious diseases.

Ebola virus disease (EVD) outbreaks in the Democratic Republic of Congo (DRC) and West Africa and the COVID-19 pandemic highlight the importance of understanding community reactions to infectious disease outbreaks [2-5]. Preparedness and response activities must suit people’s demands and cultural needs. One approach that allows integrating social sciences into antimicrobial resistance (AMR) or infectious disease research is community engagement (CE).

Developing appropriate models for multi-layered, multi-sectored, dialogue-based engagement represents one pillar of SoNAR-Global. Consequently, we performed a mapping and assessment exercise in 2019 as part of the project's first steps, searching for existing models of CE targeting infectious threats and AMR. We compared the identified examples with the UNICEF Communication for Development (C4D) Minimum Quality Standards for Community Engagement [6], which defines and narrows down the various understandings of CE and provides practical guidance for CE-based research and response activities, stressing that CE needs to be specific, localised, responsive, and bi-directional, and that top-down approaches must be avoided.

## WHAT IS COMMUNITY AND COMMUNITY ENGAGEMENT?

Current literature defines community and community engagement in different ways. What the term “community” encompasses can differ depending on a project’s objective, and its scope ranges from geographic contexts to shared interests and social and political networks. In practice, “engagement” can be defined as a “two-way dialogue between crisis-affected communities, humanitarian organizations, and (...within and between communities (enabling) affected people to meet their different needs, address their vulnerabilities, and build on their pre-existing capacities” [7].

The UNICEF Minimum Quality Standards for CE frames “communities” as wider networks that influence “the transfer of health, educational, social, informational, economic, cultural and political resources” and often include “unequal distributions of authority, access, and power over decision-making and resources” [6]. “Community engagement” is “foundational (…) for working with traditional, community, civil society, government, and opinion groups and leaders.” It is supposed to “empower(s) social groups and social networks” by building upon “local strengths and capacities” and improving “local participation, ownership, adaptation, and communication” [6]. CE strategies ideally allow all stakeholders “access to processes for assessing, analyzing, planning, leading, implementing, monitoring, and evaluating actions, programs, and policies that will promote survival, development, protection, and participation” [6]. We consider these definitions of community and CE as a practical working basis, as they “offer a common language for understanding community engagement and framing the engagement process in the context of global public health priorities” [8].

## Exploring existing engagement structures in three countries

As a SoNAR-Global partner, the Medical University of Vienna coordinated data collection during March and June 2019 with partner organisations in three countries: BRAC University of Dhaka, Bangladesh, Makerere University of Kampala, Uganda, and the Public Health Centre of the Ministry of Health of Ukraine, Kyiv.

We broadly searched for health-related interventions, programmes, or projects on AMR or infectious diseases involving communities and seeking community feedback for programme interventions [9] to gather online resources and information on CE activities that were solely available locally. The aim was to understand the nature of these interventions: e.g. who initiated the campaign? What was the purpose of the CE project? Who was the involved public? Additionally, we looked at the form of inclusion and participation, the projected outcomes, and how monitoring and evaluation instruments were integrated into the CE.

Regular online meetings were held for a continuous feedback and reflection process. The final step was a consultation meeting in June 2019, additionally involving experts from the World Health Organization (WHO), Global Research Collaboration for Infectious Disease Preparedness (GloPID-R), and the Social Science in Humanitarian Action Platform (SSHAP).

## LOCAL CHOICES

Bangladesh, Uganda, and Ukraine were chosen for the exercise because they faced multiple health challenges listed among the WHO-identified threats to global health [10] and witnessed epidemic outbreaks before and during the data collection period.

In Bangladesh, antimicrobial resistance (AMR) is a significant health issue resulting from the misuse of antibiotics in the animal farming industry and improper environmental practices [11]. In humans, the abuse of antimicrobials is associated with a weak regulatory regime, the economic interests of the pharmaceutical industry, and the demand by patients to avoid doctors’ fees [12]. In Uganda, we focused on projects tackling AMR or common viral haemorrhagic fevers (VHF) outbreaks, especially the EVD, inside the country and in the neighbouring Democratic Republic of Congo. In Ukraine, we focused on infectious-disease-related CE projects, In 2018, the country struggled with a measles outbreak including more than 54 000 cases. A lack of vaccination coverage for preventable diseases coincides with vaccine hesitancy among parents and health workers, fuelled by media anti-vaccination campaigns [13].

The SARS-CoV-2 virus was still unknown during the data collection period in 2019. However, numerous successful COVID-19-related CE activities in Uganda or Bangladesh were later conducted [14-16] (see also EU project SoNAR-global deliverables, not yet published).

We collected information on 41 projects; eleven aligned with the UNICEF Minimum Standards for CE – four in Bangladesh, four in Uganda, and three in Ukraine. Nevertheless, all projects lacked some elements of participation and two-way communication or did not conclusively define “community”. Here we present one example for each country.

In Bangladesh, the project Community Dialogue to address antibiotic resistance explored the potential of the community dialogue approach (CDA), strongly reliant on social and behaviour change theories, to improve antibiotic use on a community level [17]: the results of a qualitative study on perceptions about antibiotics and a household knowledge, attitude, and practices (KAP) survey were used for developing critical messages on antibiotics for CE activities. Community volunteers were trained in appropriate antibiotic use and AMR facts and later performed community meetings on these topics. The project sought to understand the local context by involving community members. The CDA approach included community volunteers on all project levels to facilitate community meetings and transfer knowledge. Furthermore, the study highlighted the need to hold regular feedback meetings with community volunteers to reinforce key messages and continuously document progress and setbacks for monitoring and evaluation purposes.

In Uganda, the Emergency Plan of Action for EVD Preparedness stood out [18]. It involved CE through risk communication and sensitisation. Community-based surveillance and feedback mechanisms (including a rumour-tracking system) were established. Community-based volunteers were trained in risk communication, social mobilisation, and psychological first aid techniques and carried out interpersonal communications and hygiene promotion at household and community levels, reaching almost 700 000 individuals with critical messages on EVD prevention [18]. Communities were included in all preparedness- and response pillars; however, the CE interventions in Uganda provided limited possibilities for a two-way dialogue with communities, instead relying primarily on top-down communication. Monitoring, evaluation, and learning components also played a subordinate role – certainly owed to the emergency context of these EVD projects.

In Ukraine, the CE method of Public consultations on health policy formation and implementation, which became legally binding for new legislation in 2010, was used to address growing vaccine hesitancy and the resulting measles outbreak [19-22]. Stakeholders consider the public consultation procedures transparent and sustainable, as the state statutorily funds them. The processes can be initiated by community requests or government and public institutions and should facilitate national decision-making, collaboration, and community participation on multiple levels. The consultations can occur via face-to-face meetings, electronically, or as social research. For instance, drafts of public health-related bills need to be published on a government webpage so that community members can comment on them and add suggestions for improvement. The drafts are modified only if experts endorse these suggestions, meaning public opinion is not always adopted. Systematic, formative evaluation processes have yet to be established.

## CONCLUSIONS

On a positive note, social science instruments are already used in various ways in infectious disease and AMR issues projects. Qualitative surveys, perception studies, and community dialogues were deployed in projects across our partner countries. There is a fundamental disposition to cooperate and communicate with people affected by infectious threats and an apparent effort to build on local capacities for data collection and disease sensitisation. However, at the design level, most projects deviated from the inclusion and ownership standards formulated in the UNICEF Minimum Quality Standards guidelines [6]; two-way communication models were rarely used, and projects lacked evaluation or transparency.

One fundamental challenge in applying UNICEF quality standards is its broad definition of “community”. While societal inequalities are acknowledged, it does not guide how to tackle diversity, fragmentation, or dissent within communities. We argue that this diversity becomes particularly relevant during a health crisis: outbreaks of infectious diseases can create new communities of vulnerable individuals or vulnerable stakeholders such as caregivers, mentally challenged individuals, or parents of children suffering from illness [23]. These groups do not coincide with the UNICEF guidelines’ definition of community; they are not necessarily organised in networks but represent communities of interest, often without structures or representation, especially in the early phases of infectious disease outbreaks. In our view, and considering the recent research experiences on COVID-19 of the SoNAR-Global network [24], CE should be informed by thorough assessments of vulnerabilities, considering a community’s “history and situatedness” and inherent “systems and structures” [8]. Furthermore, monitoring and evaluation of infectious diseases must be done despite the related challenge – as in all disaster and humanitarian crisis intervention contexts.

Communities of all sorts could profit from additions to the well-developed UNICEF document, which was written for community engagement in general. Considering the recent experiences of the COVID-19 pandemic, more attention should be paid to the specific circumstances in connection with infectious threats. Refining the UNICEF Minimum Quality Standards for CE accordingly would increase its impact in practice.
